# A Double-Blind, Randomized Controlled Pilot Trial to Assess the Analgesic Efficacy of Ultrasound-Guided Preemptive Caudal Morphine as an Adjunct to Bupivacaine for Lumbosacral Spine Surgeries in Adults

**DOI:** 10.7759/cureus.27647

**Published:** 2022-08-03

**Authors:** Amit K Malviya, Chhavi Sawhney, Dalim K Baidya, Sulagna Bhattacharjee, Ajeet Kumar, Kamran Farooque, Mahesh Arora, Anjolie Chhabra

**Affiliations:** 1 Department of Anaesthesiology, Pain Medicine and Critical Care, All India Institute of Medical Sciences, New Delhi, New Delhi, IND; 2 Department of Anaesthesiology, All India Institute of Medical Sciences, Patna, Patna, IND; 3 Department of Orthopaedics, All India Institute of Medical Sciences, New Delhi, New Delhi, IND; 4 Department of Anaesthesiology, Institute of Liver and Biliary Sciences, New Delhi, IND

**Keywords:** multimodal analgesia, ultrasound guided caudal, postoperative pain, preemptive analgesia, morphine, lumbosacral spine surgeries, bupivacaine

## Abstract

Background

The analgesic efficacy of preemptive administration of caudal morphine for spine surgeries in adults is not well studied. In a double-blinded, randomized controlled trial, safety and analgesic efficacy of preemptive, single-shot caudal morphine and bupivacaine was compared with caudal bupivacaine alone in lumbosacral spine surgeries.

Methods

After Institutional Ethics Committee approval, 40 patients aged 18-60 yrs planned for lumbosacral spine surgery were randomized to groups of 20 patients each. After induction and prone positioning, an ultrasound-guided caudal block was performed with morphine 50 µg/kg with 20 ml 0.25% bupivacaine in the study group (LM) and only bupivacaine in the control group (LA). Postoperatively, both groups received intravenous morphine via patient-controlled analgesia (PCA) pump (No basal, 1 mg/bolus, 10 minutes lockout interval). Intraoperative fentanyl use, postoperative 24-h morphine consumption, visual analogue pain scores (VAS) and adverse effects of morphine were noted.

Results

Demographics and baseline data were comparable. Postoperative 24-hour morphine requirement was more in LA group (34.3 ± 10.7 mg vs 19.65 ± 11.8 mg, p=0.0001). Total intraoperative supplemental fentanyl requirement was similar (79.25 ± 67.60 µg in LA vs 54 ± 50.20 µg in LM group, p=0.28). VAS scores at 2/4/6/12-hour in group-LM were significantly less than group-LA (p=0.005, 0.002, 0.001 and 0.047) but were comparable at 18 and 24 hours (p=0.25, 0.42). Postoperative incidence of adverse effects of morphine was comparable.

Conclusions

Ultrasound-guided, single-shot preemptive administration of caudal morphine with bupivacaine is a safe and effective modality of analgesia for patients undergoing lumbosacral spine surgeries.

## Introduction

Spine surgeries are associated with a high prevalence of preoperative pain and analgesic use, limited mobility, delayed recovery of pulmonary function and perioperative morbidity [1,2]. A multimodal approach to analgesia with intravenous opioids and regional techniques has been recommended for spine surgeries to reduce opioid consumption and provide a better quality of analgesia [2,3]. However, presently there is no consensus on a well-structured approach to multimodal analgesia in spine surgeries [4].

Preemptive analgesia prevents central sensitization and has been shown beneficial in spine surgeries [5]. Drugs administered in the caudal block prior to surgical incision provide good preemptive analgesia in lumbosacral spine surgeries in adults by adhering to the nerve roots, blocking CNS plasticity and prolonging analgesia [6]. The effect is potentiated by the addition of opioids as adjuvants to local anesthetics [1].

The present study was designed as a double-blind, pilot, randomized controlled trial to compare the efficacy of ultrasound-guided preemptive caudal local anesthetics administered alone or with morphine for perioperative analgesia in lumbosacral spine surgeries. We hypothesized that caudal administration of morphine as an adjuvant to local anesthetics shall be more efficacious for perioperative pain relief compared to caudal local anesthetics administered without an adjuvant. The goal of the study was to explore the effectiveness and safety of caudal morphine in spine surgeries among adults. The primary objective was to study the postoperative morphine consumption by patient-controlled analgesia (PCA) pump over 24 hours. Secondary objectives included intraoperative analgesia requirement, postoperative visual analogue scores (VAS) and postoperative adverse effects related to morphine (respiratory depression, pruritus, nausea, and vomiting).

This article was previously presented as an abstract at the European Society of Regional Anesthesia, September 11-14, 2019.

## Materials and methods

Study setting and population

**Figure 1:**
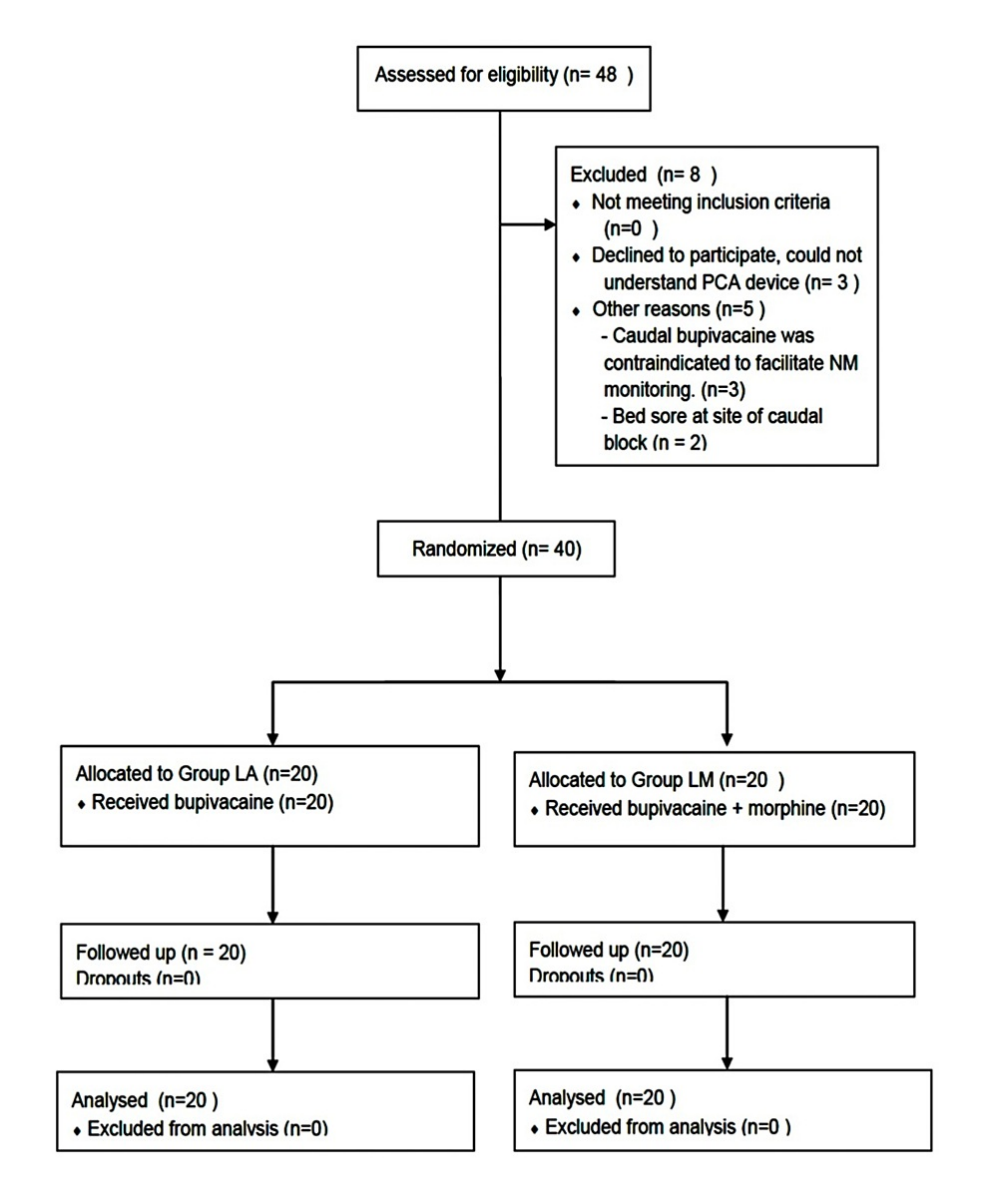
CONSORT diagram PCA: Patient-Controlled Analgesia

This study was conducted from April 2016 to July 2017, at a tertiary care multi-speciality hospital in India. The study was designed as a prospective, parallel-group, randomized, blinded, controlled clinical pilot trial designed in accordance with the Consolidated Standards of Reporting Trials (CONSORT) 2010 guidelines (Figure 1).

The study population included a total of 40 patients of the American Society of Anesthesiologists (ASA) physical status I and II of either sex, aged 18-60 years undergoing lumbosacral spine surgery (trauma and degenerative pathologies). Exclusion criteria had patients with a head injury, raised intracranial pressure, coagulopathy, infection at a local site, chronic opioid use, and allergy to the local anesthetic drug.

Ethics approval, patient consent and trial registration

All procedures followed were in accordance with the ethical standards, after obtaining Institutional Ethics Committee approval (IECPG/215/24.02.2016, RT-29/30.03.2016). Registration with the Clinical Trial Registry of India (CTRI) was obtained with trial no. CTRI/2017/11/010581 (accessible on http://ctri.nic.in/Clinicaltrials/login.php). All subjects/patients (or legal representatives) provided informed written consent prior to their recruitment into the study.

Randomization, allocation, and blinding

Patients were randomly allocated to group A (group LA) and group B (group LM) using block randomization, done with a computer-generated blocked randomization list (block size 4). Allocation concealment was done by the sealed opaque envelope technique. Informed written consent was obtained in the language of patient’s understanding prior to recruitment into the study. Opening of envelopes and preparation of the caudal drug according to the allotted group was done by an independent anesthesiologist, who neither assessed any intraoperative or postoperative outcome nor was part of the anesthesia management team.

Caudal drug mixtures were prepared with all aseptic precautions by mixing 10 ml of 0.5% bupivacaine with 8-10 ml of normal saline with or without the addition of morphine, to produce a resultant volume of 20 ml in both groups. Patients were randomly allocated to receive ultrasound-guided caudal epidural block with either 20 ml of 0.25% bupivacaine in Group LA or with morphine 50 µg/kg (maximum 3 mg) in 20 ml of 0.25% bupivacaine in Group LM. This was done to ensure that, intraoperative and postoperative outcome assessors were blinded to the group allocation. The postoperative outcome assessor was blinded to the group allocation.

Interventions in the allocated groups

Preanesthetic check-up was done and informed written consent was obtained from all patients before surgery. The patients were familiarized with the Visual Analogue Scale (VAS) and the use of patient-controlled analgesia (PCA) device. All the patients received premedication with alprazolam 0.5 mg orally at night prior to the day of surgery and two hours before surgery.

In the operating room (OR), electrocardiography (ECG), noninvasive blood pressure (NIBP), end-tidal carbon dioxide (EtCO2) and arterial oxygen saturation (SpO2) were attached. Anesthesia was induced with propofol 2 mg/kg, fentanyl 2 µg/kg and atracurium 0.5 mg/kg used to achieve neuromuscular blockade.

The trachea was intubated with an appropriate size cuffed endotracheal tube. Anesthesia was maintained with nitrous oxide (N2O) in oxygen (50:50) with Isoflurane to attain a minimal alveolar concentration of 0.8-1.0 for given age (MACage). Then, the patients were turned prone and sacral ultrasound was performed using Sonosite (S Nerve) ultrasound machine (Sonosite Inc., Bothell, WA, USA). A linear transducer probe (curvilinear for obese patients) was used to identify the relevant structures. In a transverse view of the sacrum at the level of sacral hiatus, sacral cornua were visualized as U-shaped hyperechoic structures on either side of the midline and sacrococcygeal ligament was seen as a hyperechoic band connecting them. At this position, the probe was rotated to obtain a longitudinal view and sacral hiatus was identified. A 100-mm, 22-gauge insulated nerve block needle (Stimuplex A, B. Braun Melsungen AG, Germany) was passed under vision in in-plane view and advanced under sonographic guidance through the sacrococcygeal ligament into the epidural space of sacral canal. A distinct pop could be felt as the needle penetrates the sacrococcygeal ligament. After negative aspiration, the injection was carried out under real-time ultrasonography. Dilatation of space was noted to confirm the successful spread of the drug in caudal epidural space.

Tranexamic acid (10 mg/kg bolus, followed by 1 mg/kg/hour infusion) was given routinely to all patients undergoing spine surgeries in both groups to reduce intraoperative blood loss. If there was an increase in heart rate and/or blood pressure of more than 20% from the baseline, fentanyl 0.5 µg/kg was given after ensuring adequate relaxation and normovolemia. All patients received an intravenous infusion of paracetamol (1 g) routinely, towards the end of surgery. No other intravenous analgesics except paracetamol and fentanyl were administered intraoperatively. Thirty minutes before extubation, an injection of ondansetron (4 mg) was administered to prevent postoperative nausea and vomiting (PONV). After completion of the surgery, patients were repositioned to supine, neuromuscular blockade was reversed with neostigmine (50 µg/kg) and glycopyrrolate (10 µg/kg) and the trachea was extubated.

In the post-anesthesia care unit (PACU), monitoring of vitals was done for all patients and they were assessed for pain, respiratory depression, pruritus and PONV. Baseline VAS was recorded and IV PCA morphine was attached (no basal infusion, 1 mg bolus with lockout period of 10 minutes, 4 hours limit 20 mg). For addressing postoperative pain, the caudal block was considered as the primary analgesia, paracetamol 1 g IV q8 hourly was administered as secondary analgesia, and patients were provided with PCA pump for the administration of intravenous morphine as rescue analgesia. Patients with unfulfilled analgesia needs with these modalities were excluded from the study for alternative analgesic plans. Patients were kept in a recovery room for 24 hours. Patients with episodes of vomiting received metoclopramide 10 mg IV. In case of severe pruritus, 10 mg of pheniramine maleate was administered intravenously.

Outcome measures

Data collected included postoperative 24-hour total morphine consumption as the primary outcome. Morphine consumption in 6 hourly slots was also recorded.

Demands that were fulfilled by PCA were considered as delivered demands (equivalent to total morphine consumption), demands undelivered were considered as bad demands, and a sum of both filled and unfulfilled demands constituted total morphine demands. Secondary outcomes included total intraoperative fentanyl requirement, time to the first activation of PCA, VAS scores at immediate postoperative period, 2 hours, 6 hours, 12 hours, 18 hours and 24 hours, and incidence of adverse effects (respiratory depression, pruritus, PONV).

Sample size and statistical analysis

An extensive search of the literature on PubMed, Embase and Scopus at the time of designing study (2016), at the time of statistical analysis (2018) did not reveal any study on the use of caudal morphine for lumbosacral spine surgery. Based on the existing data in our institutional practice, we anticipated 24 h morphine consumption of 30+/-10 mg (mean+-SD) in adult patients receiving caudal local anesthetic for lumbosacral spine surgeries. We anticipated approximately 30% reduction of cumulative 24 h analgesic consumption, with the addition of morphine (50 mcg/kg) as an adjuvant in the caudal block with local anesthetics. Based on this data, a total sample size of 38 patients was calculated, with alpha of 0.05 and power of 80%. Therefore, it was decided to recruit 20 samples in each group on a pilot basis.

Data were entered in spreadsheets and analysis was done using StataCorp (2015), Stata Statistical Software, Release 14 (StataCorp LP, College Station, TX). Mean, median, range, standard deviation, confidence interval and p-value were calculated using the Two-sample t-test, Chi-square test, Fisher’s exact test and Two-sample Wilcoxon rank-sum (Mann-Whitney) test. Generalized estimating equations (GEE) were used for the analysis of pain scores (VAS), morphine consumption and PCA pump demands over the 24 h period of follow-up.

## Results

A total of 48 patients were screened for eligibility, of which eight patients were excluded. Demographic and baseline data of patients were comparable (Table 1).

**Table 1:** Variables in patient characteristics, disease characteristics and surgery characteristics. Group LA – Local anesthetic, Group LM – Local anesthetic with morphine, SD – Standard deviation, Kg – kilogram, TLIF – Transforaminal Lumbar Interbody Fusion, VAS – Visual Analogue Scale, min – Minimum, max – Maximum.

Parameters	GROUP LA (n=20)	GROUP LM (n=20)	P value
Age (years, Mean ± SD)	34.3 ± 13.6	36.9 ±13.2	0.551
Weight (Kg, Mean ± SD)	59.7 ± 10.4	64.4 ± 11.2	0.176
Sex M/F (frequency, %)	13/7 (65/35)	11/9 (55/45)	0.748
ASA status (frequency, %)			
1	18 (90)	16 (80)	0.661
2	2 (10)	4 (20)	
Motor loss (frequency, %)			
Yes	7 (35)	6 (30)	0.99
No	13 (65)	14 (70)	
Sensory loss (frequency, %)			
Yes	7 (35)	6 (30)	0.99
No	13 (65)	14 (70)	
Urinary retention (frequency, %)			
Yes	7 (35)	7 (35)	0.99
No	13 (65)	13 (65)	
Level of surgery (frequency, %)			
L3 and above	8 (40)	7 (35)	0.99
L4 and below	12 (60)	13 (65)	
Disease group -(frequency, %)			
Trauma	11 (55)	11 (55)	0.99
Disc prolapse	4 (20)	5 (25)	
Degenerative	5 (25)	4 (20)	
Surgery group -(frequency, %)			
Posterior Instrumentation	11 (55)	11 (55)	0.99
Discectomy	4 (20)	5 (25)	
TLIF	5 (20)	4 (20)	
Preoperative Pain score (VAS, Median (min-max))	2 (0-3)	2 (0-3)	0.649
Duration of disease (Days, Median (min-max))	26 (1-520)	23 (1-130)	0.576
Duration of anaesthesia (Minutes, Median (min-max))	262 (135-450)	277.5 (135-480)	0.724
Duration of Surgery (Minutes, Median (min-max))	160 (75-340)	190 (75-360)	0.490

Total intraoperative supplemental fentanyl requirement was similar (79.25 ± 67.60 µg in LA vs 54 ± 50.20 µg in LM group, p=0.28). Postoperative 24-hour morphine requirement was more in LA group (34.3 ± 10.7 mg vs 19.65 ± 11.8 mg, p=0.0001). Total 24 h morphine demands, and bad demands were significantly more in LA group. However, the mean time to first PCA demand was comparable between the groups (12 ± 17.9 minutes in LA group and 24 ± 43 minutes in LM group, p=0.7) (Table 2).

**Table 2:** Intraoperative and postoperative analgesia Group LA – Local anesthetic, Group LM – Local anesthetic with morphine, SD – Standard deviation, IQR – interquartile range, µg – microgram, h – hours, mg – milligram, PCA – patient-controlled analgesia

Parameter	GROUP LA (n=20)	GROUP LM (n=20)	P value
Intraoperative supplemental fentanyl (µg), mean (SD)	79.25 (67.60)	54 (50.20)	0.2804
Cumulative 6 h morphine consumption (mg), mean (SD)	13.85 (5.04)	7.10 (3.71)	
Cumulative 6 h morphine consumption, median (IQR)	15.00 (10.50, 16.50)	7.50 (4.50, 10.00)	<0.001
Cumulative 12 h morphine consumption, mean (SD)	20.80 (5.72)	11.45 (6.25)	
Cumulative 12 h morphine consumption, median (IQR)	21.50 (18.00, 23.50)	11.50 (6.00, 15.50)	<0.001
Cumulative 18 h morphine consumption, mean (SD)	27.95 (7.42)	15.35 (8.74)	
Cumulative 18 h morphine consumption, median (IQR)	28.00 (25.00, 34.50)	15.00 (9.00, 21.00)	<0.001
Total 24 h morphine consumption, mean (SD)	34.30 (10.70)	19.65 (11.18)	
Total 24 h morphine consumption, median (IQR)	32.00 (28.50, 41.50)	20.00 (10.50, 28.50)	<0.001
Cumulative 6 h PCA morphine demands, mean (SD)	44.35 (38.60)	12.75 (8.93)	
Cumulative 6 h PCA morphine demands, median (IQR)	31.50 (24.00, 60.50)	10.50 (4.50, 20.50)	<0.001
Cumulative 12 h PCA morphine demands, mean (SD)	58.55 (38.45)	21.70 (16.15)	
Cumulative 12 h PCA morphine demands, median (IQR)	49.00 (37.50, 76.00)	18.00 (9.00, 32.00)	<0.001
Cumulative 18 h PCA morphine demands, mean (SD)	75.70 (43.52)	28.80 (21.96)	
Cumulative 18 h PCA morphine demands, median (IQR)	63.50 (45.00, 100.50)	23.50 (11.50, 46.00)	<0.001
Total 24 h PCA morphine demands, mean (SD)	87.10 (48.51)	37.65 (28.93)	
Total 24 h PCA morphine demands, median (IQR)	77.50 (51.00, 112.50)	32.00 (13.50, 56.50)	<0.001
Cumulative 6 h bad demands, mean (SD)	30.50 (35.57)	5.65 (5.81)	
Cumulative 6 h bad demands, median (IQR)	17.50 (11.50, 42.00)	4.00 (0.50, 10.00)	<0.001
Cumulative 12 h bad demands, mean (SD)	37.75 (35.28)	10.25 (10.72)	
Cumulative 12 h bad demands, median (IQR)	27.00 (17.50, 51.00)	6.50 (2.50, 18.00)	<0.001
Cumulative 18 h bad demands, mean (SD)	47.75 (38.48)	13.45 (14.18)	
Cumulative 18 h bad demands, median (IQR)	38.50 (18.00, 65.00)	8.00 (2.50, 23.00)	<0.001
Total 24 h bad demands, mean (SD)	51.80 (40.26)	18.00 (19.34)	
Total 24 h bad demands, median (IQR)	47.00 (20.50, 71.00)	10.50 (3.00, 31.50)	0.002

**Figure 2:**
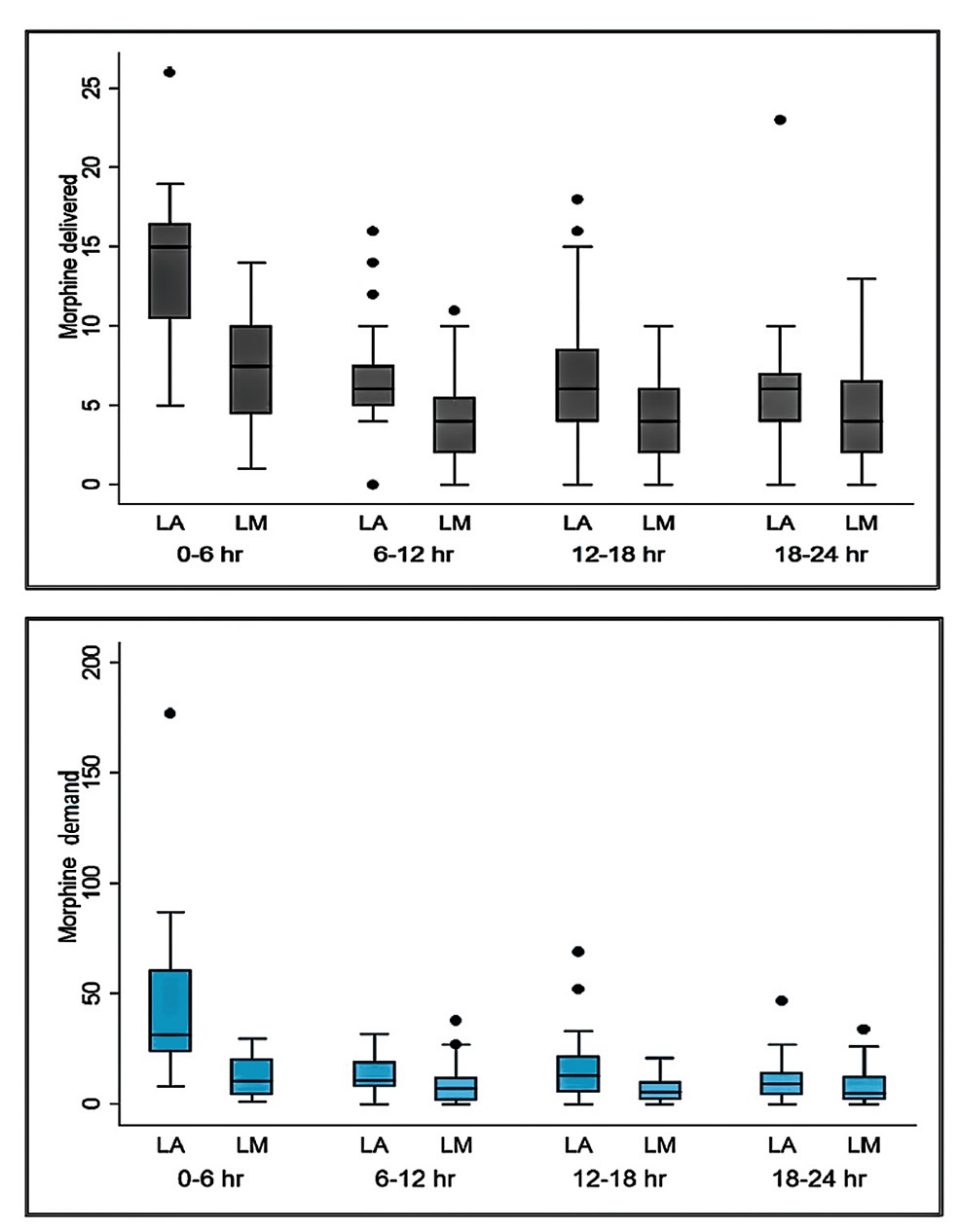
(a) Total number of PCA morphine delivered; (b) Total number of PCA morphine demands Group LA – Local Anesthetic Alone, LM – Local Anesthetic with morphine, PCA – Patient-controlled analgesia

Morphine consumption was significantly lower in group LM as compared to group LA during the time slots of 0-6 h, 6-12 h and 12-18 h (p values- 0.0001, 0.009 and 0.022, respectively). PCA demands were significantly lower in group LM as compared to group LA during the time slots of 0-6 h, 6-12 h and 12-18 h (p values- 0.0001, 0.028 and 0.022, respectively). Both morphine consumption and demands were comparable after 18 h (p=0.13, 0.25) (Figure 2).

Preoperative visual analogue pain score (VAS) was comparable between the two groups (1.95 ± 0.99 in Group LA vs 1.8 ± 1.06 in Group LM, p=0.65). In the immediate postoperative period VAS scores in the two groups (4.65 ± 2.27 in LA, 3.75 ± 1.99 in LM ) were comparable (p-value 0.22). VAS scores were significantly less in Group LM at 2, 4, 6 and 12 hours (p-value 0.005, 0.002, 0.001 and 0.047 respectively). However, VAS scores at 18 hours (3.7 ± 1.45 in LA vs 3.05 ± 1.19 in LM) and 24 hours (3.5 ± 1.31 in LA vs 3.5 ± 1.31 in LM) were comparable (p-value = 0.25 and 0.42).

Statistical analysis was also done by Generalized Estimating Equation (GEE) to evaluate the effect of group (LA and LM) and time (hours) on pain scores at rest and motion, and total, delivered, and undelivered PCA morphine demands. It was found that morphine consumption, PCA demands and VAS scores at rest and motion were significantly different for groups and time (Table 3).

**Table 3:** GEE estimated pain scores, morphine consumption and PCA demands GEE – Generalized estimating equations, PCA – Patient-controlled analgesia, VAS – Visual analogue scale for pain score. [PCA total demands, undelivered demands (bad demands) and delivered demands (morphine consumption) were recorded at 6, 12, 18 and 24 h postoperatively. VAS for pain at rest and motion were recorded at baseline preoperatively and 0, 2, 6, 12, 18 and 24 h postoperatively.]

S. No	Parameter	Effect	Coefficient	95% confidence interval	P value
1. a	PCA delivered demands (Morphine consumption)	Group	-3.6375	-5.291855 to -1.983145	<0.0001
1. b	PCA delivered demands (Morphine consumption)	Time	-1.5725	-2.092245 to -1.052755	<0.001
2. a	PCA total demands (delivered + undelivered)	Group	-12.35	-18.38761 to -6.312389	<0.001
2. b	PCA total demands (delivered + undelivered)	Time	-5.51	-7.932194 to -3.087806	<0.001
3. a	PCA bad demands (undelivered)	Group	-8.7125	-13.51332 to -3.911679	<0.001
3. b	PCA bad demands (undelivered)	Time	-3.9375	-6.042104 to -1.832896	<0.001
4. a	VAS at rest	Group	-0.725	-1.289979 to -0.160021	0.012
4. b	VAS at rest	Time	-0.1507143	-0.238668 to -0.06276	0.001
5. a	VAS at motion	Group	-0.6833333	-1.333749 to -0.0329176	0.039
5. b	VAS at motion	Time	-0.1571429	-0.257954 to -0.0563317	0.002

There was no significant difference in the incidence of adverse effects (PONV, pruritis) between the two groups. The incidence of PONV was 40% in group LA, and 45% in group LM. The incidence of pruritus in group LA was 45% and it was 50% in group LM. Respiratory depression was not observed in any patient.

## Discussion

In this study, single shot caudal bupivacaine (0.25%, 20 ml) administered along with morphine (50 µg/kg) prior to spine surgery, significantly reduced postoperative intravenous morphine requirement and PCA pump demands, as well as postoperative pain scores (VAS) without an increase in adverse effects, compared to the group receiving bupivacaine without adjuvant. There was no significant difference in intraoperative fentanyl requirement, and mean time to first PCA demand. Ultrasound use with real-time visualization of caudal administration of drug appeared to be a useful and safe technique in the adult patient population.

Caudal morphine (50 µg/kg) administered preemptively along with bupivacaine (0.25%, 20 ml) reduced postoperative analgesic requirement more effectively as evidenced by significantly lower VAS score and morphine consumption via PCA device in bupivacaine with the adjuvant group. Previous studies investigating pain relief after lumbar spine surgery, have found decreased postoperative analgesia requirement with caudal administration of 20-30 ml of local anesthetics (bupivacaine and ropivacaine, 0.166% to 0.375%) with or without adjuvants [6-10]. Buprenorphine (0.1 mg), tramadol (50 mg), ketamine, dexamethasone (8 mg) and dexmedetomidine (1 µg/kg) have been used effectively as adjuvants without an increase in adverse effects [6-10]. While the role of caudal morphine as an adjuvant to local anesthetics for postoperative analgesia has not been studied for spine surgery, it has been found to be effective for genital surgeries in doses of 30-100 µg/kg [11]. We decided to assess the analgesic efficacy of preemptive caudal morphine (50 µg/kg) as an adjuvant to 20 ml of 0.25% bupivacaine and found it to be more effective than caudal bupivacaine administered alone.

The mean time to first analgesic demand was less than 30 minutes in both the groups. Most of the previous studies observed this time to be as high as 10 hours. One of the confounding factors could be the intraoperative use of long-acting analgesics in previous studies like intramuscular diclofenac [6], intravenous dexamethasone [8], fentanyl infusion [10], or intravenous morphine. We did not administer any intraoperative analgesia except fentanyl (2 µg/kg) at the time of induction. Another potential explanation for these observations could be the surgical characteristics. Most of the previous studies included discectomy or laminectomy [9-10] or only a few patients (20%) underwent instrumentation [8]. Whereas in the present study, most patients had traumatic pathology (55%) and 31 out of 40 (78%) underwent instrumentation.

In our study, the analgesic administration was by patients’ choice via PCA pump, which is superior to intermittent divided dosing, in terms of analgesia and ease of use [2].

Intraoperative fentanyl requirement was comparable between the groups (p=0.28), which was possibly because the duration of action of caudal bupivacaine (120 to 240 minutes) corresponded with the duration of surgery (150 to 180 minutes) in both groups.

In both the groups, preoperative VAS scores were similar and therefore did not influence the difference in postoperative VAS scores or analgesic consumption. The VAS scores were significantly less in LM group from 2 hours up to 12 hours post-surgery. This corresponds to and therefore, can be attributed to the duration of action of caudal morphine.

We found that there was statistically insignificant difference in the incidence of PONV in the two groups (40% and 45%). This can be attributed to more consumption of PCA morphine in LA group as compared to LM group. Reported incidence of PONV after neuraxial morphine is 60-80% [12]. The lower incidence of PONV seen in our study can be due to preoperative anxiolysis, adequate hydration, prophylactic ondansetron and postoperative oxygen therapy used as part of our study protocol, which are components of multimodal PONV prevention strategies [13,14]. Moreover, the incidence of PONV after opioid administration is less in patients with preoperative pain [13].

Incidence of pruritus was 45% in group LA and 50% in group LM which was statistically comparable. None of the patients had severe pruritus requiring administration of antihistaminics. The reported incidence of pruritus after neuraxial opioids ranges between 30 and 100% [15]. The absence of severe pruritus may be due to the use of propofol and ondansetron in the present study, which are known to reduce the incidence of itching due to neuraxial opioids [16].

Respiratory depression, as classically defined with a respiratory rate of less than 10 breaths/min, is the most dreaded adverse effect of neuraxial opioids, was not observed in any patient in the present study. Recently, a report by the American Society of Anesthesiology task force on neuraxial opioids concluded that there is no significant difference in respiratory depression for single-shot neuraxial opioids when compared with parenteral opioids [17]. All patients were monitored in the PACU for 24 hours with SpO2 and were provided supplemental oxygen by the facemask in the postoperative period.

Most trauma patients were catheterized either preoperatively due to autonomic involvement or in the OT. Thus, the incidence of urinary retention as a side effect of opioids could not be appropriately reflected in our study.

In our study, the patient population was versatile in terms of demographics, disease pathology (traumatic, degenerative and disc prolapse), extent of surgery (L1 to S2), type of surgery [posterior instrumentation, discectomy and Transforaminal Lumbar Interbody Fusion (TLIF)] and sensory-motor or autonomic involvement. On analysis of these factors, there was no significant difference in the distribution of the above factors in the two study arms.

Neuraxial opioids provide effective perioperative analgesia for spine surgery while minimizing the dose-dependent adverse effects of systemic opioids [2,18]. Although epidural catheters provide an extended and better quality of analgesia we used a single shot technique, as caudal catheter placement is difficult in patients with spine pathology and it can lead to bacterial colonization [19].

Ultrasound guidance was used in our study as it allows good visualization of landmarks, real-time visualization of drug administration and spread with higher success rates [20]. Landmark-based blind technique for the caudal block has high success rates (96%) in children [21], while in adults it has a success rate of only 68-75% [11,22-24] owing to the difficulty in localizing the sacral hiatus due to the variation in sacral size, shape and volume [25] with risk of inadvertent dural punctures [26]. Fluoroscopy has limited use despite good accuracy owing to bulkier machine, limited availability and radiation hazards [27]. Ultrasound guidance provided good visualization of the caudal space and easy needle placement in most young patients but access to caudal space was challenging with growing age due to difficulty in negotiating the needle through the ligament. However, needle placement could be achieved in all patients.

Strengths of our study include the use of preemptive single shot technique over use of postoperative catheters, use of ultrasound guidance over landmark technique, use of multimodal analgesia including caudal block and intravenous opioids and paracetamol, use of PCA pump for patient-controlled analgesia approach over intermittent divided dosing.

Limitations of our study include a small sample size, pilot study and inclusion of spine surgeries of varying extensiveness but they were equally distributed in two groups.

Morphine appears to be a promising adjuvant to caudal local anesthetics for reducing perioperative pain and systemic analgesic requirement without any increase in adverse effects, further studies are advised for its comparison with other adjuvants used for caudal administration in spine surgeries.

## Conclusions

In this randomized controlled trial, single shot preemptive caudal bupivacaine along with morphine (50 µg/kg), significantly reduced postoperative pain scores and 24-hour postoperative intravenous PCA morphine requirements compared to bupivacaine alone in lumbosacral spine surgery. However, there was no significant difference in intraoperative fentanyl requirement and adverse effects were similar.

Therefore, preoperative use of single shot caudal local anesthetic in combination with morphine should be considered in lumbosacral spine surgery. A larger randomized controlled trial may be performed to precisely demonstrate the beneficial effects further.
